# Facial Cellulitis and Skin Abscess: A Case of a Simple Bone Cyst in the Mandibular Bone

**DOI:** 10.7759/cureus.54579

**Published:** 2024-02-20

**Authors:** Katsunori Tanaka, Yasuhiko Tsutsumi, Takumi Nakatani, Midori Tagaya

**Affiliations:** 1 Department of Pediatrics, National Hospital Organization Higashi-Ohmi General Medical Center, Higashiomi, JPN; 2 Department of Oral and Maxillofacial Surgery, National Hospital Organization Higashi-Ohmi General Medical Center, Higashiomi, JPN; 3 Department of Medical Treatment, National Hospital Organization Higashi-Ohmi General Medical Center, Higashiomi, JPN

**Keywords:** mandibular imaging, simple bone cyst, odontogenic cellulitis, skin abscess, facial cellulitis

## Abstract

Cellulitis, abscess, or both are among the most common skin and soft tissue infections. Typically, cellulitis arises due to bacterial penetration through breaches in the skin's protective barrier. However, in cases of facial cellulitis, it is necessary to consider not only the breakdown of the skin barrier but also to differentiate odontogenic cellulitis. A prompt and accurate diagnosis of facial infections stemming from dental issues, coupled with the administration of antibiotics and dental interventions, played a crucial role in resolving this condition. Odontogenic cellulitis often develops as a result of dental caries. However, we experienced a case of odontogenic cellulitis and skin abscess occurring due to a simple bone cyst in the mandible, even in the absence of dental caries. Proper imaging examinations are crucial for diagnosis.

## Introduction

Cellulitis, abscess, or both are among the most common skin and soft tissue infections. The diagnosis of cellulitis and skin abscesses is usually based on clinical manifestations. Cellulitis manifests as an area of skin erythema, edema, and warmth. Skin abscess manifests as a painful, fluctuant, erythematous nodule, with or without surrounding cellulitis. Skin abscess is a collection of pus within the dermis or subcutaneous space [1,2]. Cellulitis is treated with appropriate antibiotic administration, depending on the patient's underlying conditions and state. Skin abscess is managed with proper antibiotic treatment and incision and drainage [1,2].

Generally, cellulitis develops as a result of bacterial entry via breaches in the skin barrier [2]. On the other hand, in facial cellulitis, it is necessary to consider not only the breakdown of the skin barrier but also to differentiate odontogenic cellulitis [2,3]. Odontogenic cellulitis accounted for 50% of all facial infections in the study by Biederman et al. [4,5] and almost 54% of pediatric patients with facial odontogenic cellulitis required hospitalization in the study by Kuo et al. [4,6]. Facial cellulitis of odontogenic origin is caused by an illness in the dental and periodontal structures. The main cause is dental caries that are not treated in time, which compromise the pulp, generally causing necrosis of the pulp of the affected dental structure [3]. Other causes include necrosis of the dental pulp as a result of trauma, periodontitis, or inflammation of the peri coronal tissues associated with difficult tooth eruption [4,7]. Odontogenic facial cellulitis caused by simple bone cysts is rare.

Simple bone cysts are pseudocysts that can occur in the mandible. In the field of dental and oral surgery, a simple bone cyst is often diagnosed incidentally during imaging examinations [8]. Here, we present a case of odontogenic facial cellulitis and skin abscess caused by a simple bone cyst, despite the absence of dental caries or oral lesions.

## Case presentation

A 13-year-old boy was admitted with three days of fever, facial pain, and swelling. A day prior to the onset of the fever, he had pain from the lower jaw to the left cheek. He visited two dental clinics on the first day of his fever, but no dental caries, oral lesions, or odontogenic infection was found, and the cause of the pain remained unknown. On the second day of his fever, he developed swelling extending from the lower jaw to the left cheek. He was diagnosed with facial cellulitis at a pediatric clinic, and treatment with oral cefdinir was initiated. However, his symptoms worsened and he was referred to our hospital. He did not have a history of previous hospitalizations. His vaccinations were up to date and he had no known allergies.

A clinical examination revealed the following findings: body weight, 52 kg; body height, 170 cm; temperature, 38.6℃; heart rate, 136 beats/minute; respiratory rate, 18 breaths/minute; and blood pressure, 121/74 mmHg. He had swelling from the lower jaw to the left cheek, with tenderness, erythema, and warmth. There was no trauma, eczema, or impetigo on his skin. No dental caries, swelling, or redness were observed in his oral cavity. A blood examination revealed the following: white cell count, 13.0×109/L; neutrophil count, 10.7×109/L; c-reactive protein, 86.6 mg/L; procalcitonin, 0.10 ng/ml, and erythrocyte sedimentation rate, 42 mm/hour. He was diagnosed with cellulitis of the face, and the intravenous administration of cefazolin (CEZ) was initiated.

**Figure 1:**
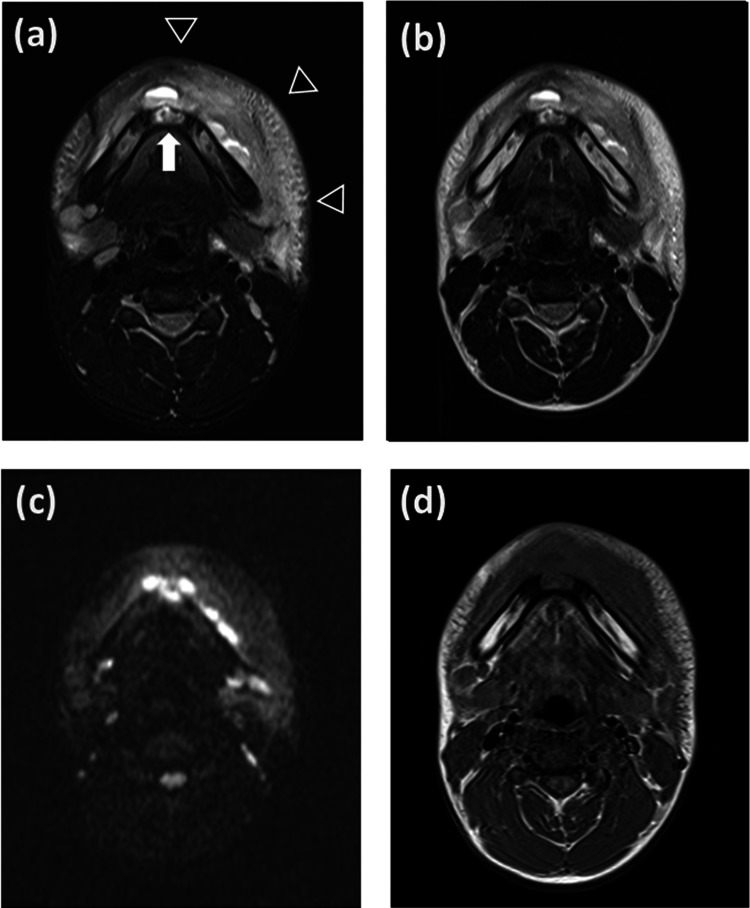
Magnetic resonance imaging. (a) Fat-suppressed T2-weighted imaging. An arrow indicates an area of high signal intensity in the middle of the mandible. Arrowheads indicate severe swelling of the subcutaneous tissue of the jaw and fluid retention along the mandibular bone. (b) T2-weighted imaging. (c) Diffusion-weighted imaging. (d) T1-weighted imaging.

**Figure 2:**
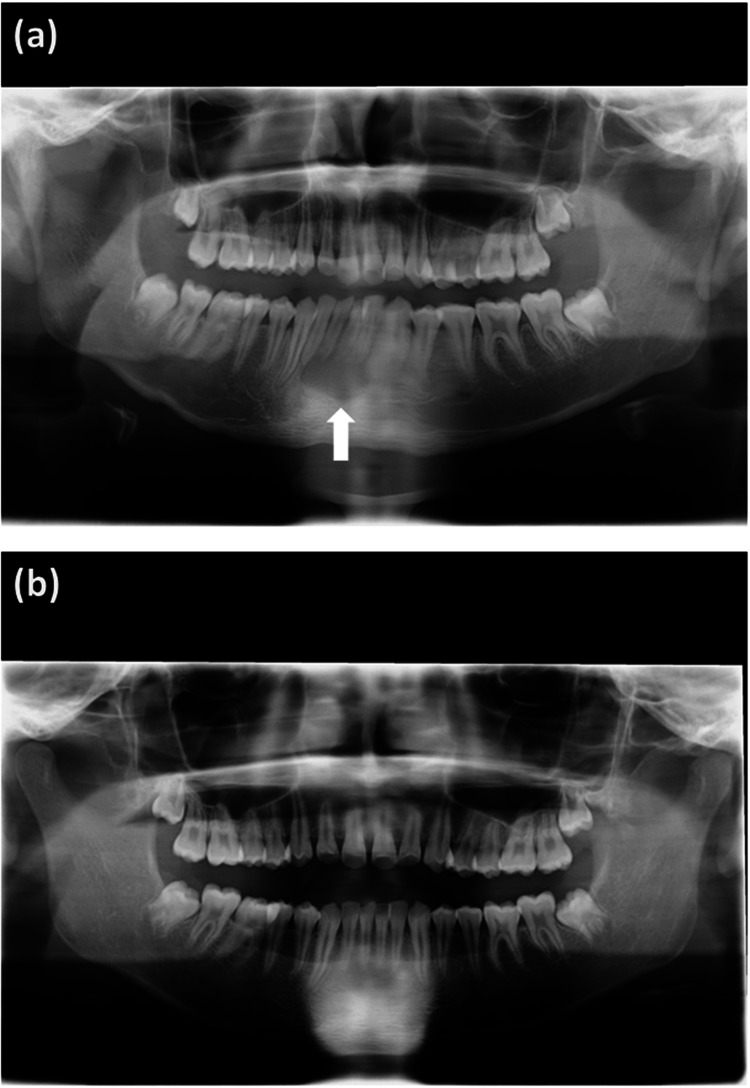
Panoramic radiographs of simple bone cyst in mandibular bone. (a) Day 3 of our treatment. (b) Eight months after our treatment.

**Figure 3:**
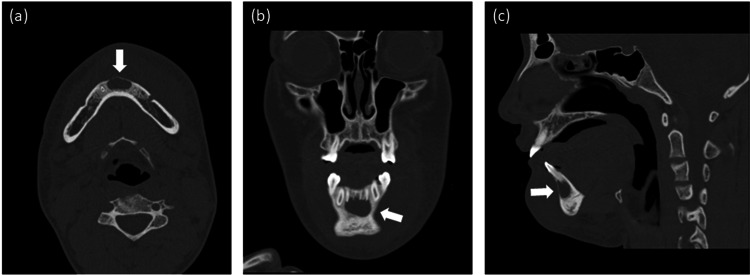
Computed tomography (a) Axial, (b) coronal, and (c) sagittal computed tomography images revealed the simple bone cyst in the mandibular bone.

Magnetic resonance imaging (MRI) was performed to evaluate the subcutaneous tissue and bone marrow to identify any complications such as osteomyelitis. MRI showed severe swelling of the subcutaneous tissue of the jaw and liquid retention along the mandibular bone, with no evidence of osteomyelitis. MRI did not clearly detect a cyst; however, it showed an area of high signal intensity in the middle of the mandible (Figure 1). A panoramic radiograph showed a cyst in the mandibular bone (Figure 2a). Computed tomography (CT) also showed the cyst at the same site (Figure 3).

We performed incision and drainage and aspirated a large amount of pus from the cyst on the fourth day of our treatment. The involved teeth showed no root resorption and were not inactivated. A histopathological examination revealed that the bone cyst did not contain an epithelial lining. On the seventh day of our treatment, the fever subsided, facial swelling resolved, and his symptoms improved. We treated him with CEZ for a total of seven days followed by cefalexin for a total of three days. He recovered without recurrence. Cultures of debrided material and blood did not identify the specific pathogen, possibly because antibiotic treatment was initiated before specimen collection. We confirmed that the bone cyst had spontaneously ossified after eight months and thus diagnosed it to be a simple bone cyst (Figure 2b).

## Discussion

In facial cellulitis, it is necessary to consider not only the breakdown of the skin barrier but also to differentiate odontogenic cellulitis. Odontogenic facial cellulitis is often caused by dental caries [3,4,7]. This case did not show any dental caries or intraoral lesions; however, odontogenic facial cellulitis and abscess developed. The simple bone cyst was the cause of facial cellulitis and abscess.

A simple bone cyst is a pseudocyst that lacks an epithelial lining, which develops in long bones, such as the humerus, femur, or mandible. The etiology of simple bone cysts is uncertain, and various hypotheses have been proposed. A simple bone cyst usually progresses slowly or does not progress at all, without swelling, pain, or any evidence of infection. In the orthopedics field, some patients with simple bone cysts complain of pain or pathological fractures. In the field of dental and oral surgery, simple bone cyst is often diagnosed incidentally during imaging examinations. Decompression or drainage has been suggested as treatment, perforating the cyst wall or creating communication with the medullary cavity [8,9]. As far as our search indicates, there are no reports of a simple bone cyst serving as a source of infection, and it is considered rare for a simple bone cyst to be the trigger for an infection as in this case. However surgical drainage may be required, as in this case. Simple bone cysts should be considered in the differential diagnosis when treating patients with facial cellulitis or abscesses.

The diagnosis of facial cellulitis should take into consideration appropriate imaging examinations. MRI is suitable for the evaluation of soft tissues and bone marrow, while radiographs or CT are suitable for the evaluation of bone lesions. In this case, a panoramic radiograph and CT were useful for diagnosing the simple bone cyst, while MRI did not clearly visualize it.

## Conclusions

When managing facial cellulitis or abscess, even in the absence of dental caries or oral lesions, one should consider the possibility of simple bone cyst in the differential diagnosis. Swift and accurate diagnosis of facial infections originating from dental problems, combined with the judicious administration of antibiotics and appropriate dental interventions, play a pivotal role in the expeditious resolution of this condition. It is recommended to conduct appropriate imaging examinations, including not only MRI but also radiography or CT scans.
